# Lipoprotein(a) in the Korean Pediatric Population Visiting Local Clinics and Hospitals

**DOI:** 10.3390/nu14142820

**Published:** 2022-07-08

**Authors:** Rihwa Choi, Sang Gon Lee, Eun Hee Lee

**Affiliations:** 1 Department of Laboratory Medicine, Green Cross Laboratories, Yongin 16924, Korea; pirate0720@naver.com; 2 Department of Laboratory Medicine and Genetics, Samsung Medical Center, Sungkyunkwan University School of Medicine, Seoul 06351, Korea; 3 Green Cross Laboratories, Yongin 16924, Korea

**Keywords:** lipoprotein(a), Lp(a), dyslipidemia, pediatric, children, Korea

## Abstract

In this paper we investigate serum lipoprotein(a), an independent risk factor for cardiovascular disease in the Korean pediatric population. Visiting local clinics and hospitals, 600 lipoprotein(a) tests were performed on 416 Korean children and adolescents (124 boys and 292 girls), with a median age of 11.1 years (interquartile range, IQR, 9.8–13.9). The median lipoprotein(a) level was 21.5 nmol/L (IQR, 8.2–51.7). Among the 416 patients, the 90th percentile value of the initial lipoprotein(a) measurement was 107.8 nmol/L. The proportion of patients with lipoprotein(a) ≥ 100 nmol/L was 11.3%. The lipoprotein(a) level and the proportion of patients with lipoprotein(a) ≥ 100 nmol/L were not significantly different among sex, or age group. Among the 416 patients, 122 (29.3%, 21 boys and 101 girls) underwent at least two follow-up lipoprotein(a) measurements. The median follow-up period was 6.7 months (IQR, 5.5–11.8). The median lipoprotein(a) level across the 122 patients was 25 nmol/L (IQR 10.0–72.0). Among those patients, seven (5.7%) experienced an increase in serum lipoprotein(a) to ≥100 nmol/L during follow-up measurements. Further studies are needed in the Korean pediatric population in order to clarify the clinical significance of this change long-term.

## 1. Introduction

Lipoprotein(a), a circulating lipoprotein composed of apolipoprotein B-containing modified low-density lipoprotein (LDL) particles covalently linked to apolipoprotein(a), has been highlighted as an independent risk factor for atherosclerotic cardiovascular dis-ease and calcified valvular diseases [1,2,3,4]. The *LPA* gene that encodes the apolipoprotein(a) component of lipoprotein(a) is associated with the circulating level of lipoprotein(a), which is 90% genetically determined [1,5,6,7,8]. The distribution of lipoprotein(a) level varies with ethnic group, with the highest 90th percentile value in those with African ancestry (199 nmol/L), followed by Caucasians (154 nmol/L). The lowest 90th percentile values were found in Japanese Americans (75 nmol/L) [7,9,10,11].

Major lipoproteins in blood, which include chylomicrons, very low-density lipoprotein (VLDL), intermediate-density lipoprotein (IDL), LDL, high-density lipoprotein (HDL), and lipoprotein(a) have been of clinical focus in the diagnosis and management of dyslipidemia [12]. A causal relationship between high plasma concentration of lipoprotein(a) and an increased risk of atherosclerotic cardiovascular disease–related events, has been strongly supported using multiple studies [6,9,10,12,13,14]. In addition, proprotein convertase subtilisin/kexin type 9 (PCSK9) inhibitors have been introduced to reduce cardiovascular risk and lipoprotein(a) level [6,9,10,11,12,15]. Thus, recent European clinical guidelines have recommended universal lipoprotein(a) screening once in an individual’s lifetime, while the National Lipid Association, in the United States, suggest targeted screening for at-risk patients [9,10]. Current clinical guidelines on dyslipidemia in Korea also recommend measurement of LDL, HDL, non-HDL (= total cholesterol–HDL), triglycerides, apolipoprotein B, and lipoprotein(a) [16].

Meanwhile, there have been inconsistencies in clinical guidelines on the measurement of lipoprotein(a) in pediatric populations, which advise either universal screening, targeted screening, or no screening [6,17]. As screening for lipids in children can lead to early identification and control of pediatric dyslipidemia, universal lipid screening with the measurement of non-fasting non-HDL in all children aged 9–11 and 17–21 years is recommended by the committee of Clinical Practice Guidelines of the Korean Society of Pediatric Endocrinology [18]. This guideline recommends measurement of non-HDL (not lipo-protein(a) itself) as it includes all atherogenic cholesterol types, including lipoprotein(a) [18]. However, causal relationships between lipoprotein(a) and events of atherosclerotic cardiovascular disease, and calcified valvular diseases, are independent of concentrations of other lipids and lipoproteins, including LDL; therefore, lipoprotein(a) measurement is recommended in western populations [3,5,6,17]. Guidelines in the 2011 National Heart, Lung and Blood Institute Expert Panel suggest measurement of lipoprotein(a) as a targeted screening in all children ≥ 2 years of age who have risk factors such as: a family history of hypercholesterolemia; lipid-lowering medications; elevated lipoprotein(a); premature cardiovascular disease; unknown risk (adopted children); a history of hypertension; increased BMI; current cigarette smoking; HDL < 40 mg/dL; Kawasaki disease; chronic inflammatory disease; human immunodeficiency virus infection; chronic kidney disease; nephrotic syndrome; diabetes; heart transplant; or unknown causes of ischemic stroke [19].

Although several analytical methods are available for measurement of lipoprotein(a), there is a lack of assay standardization [5,20,21]. It is recommended that clinicians use assays that report results in nmol/L, using a 5-point or similar calibrator, traceable to the WHO/International Federation of Clinical Chemistry and Laboratory Medicine (IFCC) secondary reference material (SRM) [3]. Unlike other lipids and lipoproteins, direct conversion between mg/dL and nmol/L is not possible (varies from 1.67 to 3.3 x conversion factor from mg/dL to nmol/L used in published data) as the proposed Reference Material 2B (IFCC SRM2B) is in nmol/L, and lipoprotein(a) isoforms have different molecular weights, due to the size heterogeneity of apolipoprotein(a) [5,20]. However, previous studies have reported lipoprotein(a) concentration with mg/dL units and conversion factors, which has caused confusion in physicians managing patients in different ethnic populations [1,5,17,21]. For example, a previous study performed with Japanese Americans using lipoprotein(a) test results, obtained by using the enzyme-linked immunosorbent assay (ELISA) method, reported 75 nmol/L as the lowest 90th percentile value, based on a conversion factor of 2.4 [5,10].

Meanwhile, large population-based studies of lipoprotein(a) levels in the Korean pediatric population are lacking; only one study with a limited number of subjects analyzed with an ELISA method has been reported [6,18,22]. Information on the intra-individual changes in quantitative serum lipoprotein(a) levels in the Korean pediatric population is, therefore, limited.

In this study, we aimed to investigate serum lipoprotein(a) test results in a Korean pediatric population in order to understand the distribution of lipoprotein(a). We also aimed to investigate intra-individual changes in serum lipoprotein(a) by considering different criteria to help physicians understand the general proportion of patients expected to undergo a change in serum lipoprotein(a) level during follow-up measurement, based on the definitions applied to patient management.

## 2. Materials and Methods

### 2.1. Subjects

We retrospectively reviewed data (obtained from the laboratory information system of Green Cross Laboratories between 1 January 2019 and 31 December 2021), for a Korean pediatric population (age < 18 years) who visited local clinics and hospitals and underwent serum lipoprotein(a) testing. Patients with missing age or sex data were excluded from the review and all data were anonymized prior to statistical analysis. To investigate intra-individual changes in serum lipoprotein(a) levels, patients who underwent at least two serum lipoprotein(a) measurements were included in the analysis.

### 2.2. Analytical Methods

Serum lipoprotein(a) was measured using an automated particle-enhanced immunoturbidimetric assay, using a Tina-quant Lipoprotein(a) Gen.2 reagent kit (Roche, Mannheim, Germany) on c702 analyzers (Roche, Mannheim, Germany). The analytical measurement range of the serum lipoprotein(a) assay was 7.0–240.0 nmol/L. The clinical reportable range with automated 1:3 dilution, for which the results were multiplied by a factor of 3, was 7.0–720.0 nmol/L. The calibrator of this assay was traceable to the IFCC reference material SRM2B.

### 2.3. Definitions

The age groups were defined as < 2 years, 2 to 4 years, 5 to 8 years, 9 to 11 years, and 12 to 17 years. The age groups were based on full expression of the *LPA* gene by 1 to 2 years of age, achievement of adult concentration of lipoprotein(a) by 5 years of age, and universal screening for lipid in pediatric populations from 9 to 11 years [5,6,18]. The proportion of patients with initial serum lipoprotein(a) <100 nmol/L (a tentative universal cut-off point recommended by the National Lipid Association in the United States) and who experienced an increase to ≥100 nmol/L during follow-up, was investigated [5]. Among patients who had follow-up serum lipoprotein(a) results, the proportion of patients who had stable serum lipoprotein(a) <100 nmol/L; who had stable increased lipoprotein(a) ≥100 nmol/L; and who had fluctuating lipoprotein(a) whether <100 nmol/L or ≥100 nmol/L, were investigated. The proportion of patients whose lipoprotein(a) level was extremely high (>430 nmol/L), and who might have increased lifetime risk of atherosclerotic cardiovascular disease similar to that of patients with heterozygous familial hypercholesterolemia, was also investigated [12].

### 2.4. Statistical Analysis

We investigated the quantitative and qualitative (high or not) results of the lipoprotein(a) test by sex and age group. The Shapiro-Wilks test was performed to test for normality of continuous variables. A non-parametric test was adopted, where appropriate, for non-normally distributed continuous variables including age; follow-up numbers; follow-up period; and serum lipoprotein(a) level (two-tailed test). Chi-square tests were used to compare the categorical variables of sex, age group, and serum lipoprotein(a) ≥100 nmol/L or >430 nmol/L. Statistical analysis was executed using MedCalc Statistical Software Version 20.110 (MedCalc Software bv, Ostend, Belgium; https://www.medcalc.org; accessed on 29 July 2022). *p* values were considered significant at the 0.05 level.

### 2.5. Ethical Approval

This study was conducted according to guidelines outlined in the Declaration of Helsinki, and all procedures involving human subjects were approved by the Institutional Review Board of Green Cross Laboratories (GCL-2022-1026-01, 3 June 2022). A waiver of informed consent was approved by the IRB as the study was retrospective and involved no more than minimal risk to subjects.

## 3. Results

### 3.1. Characteristics of Study Subjects and Baseline Serum Lipoprotein(a) 

During the study period, 600 lipoprotein(a) tests were performed on 416 Korean children and adolescents (124 boys and 292 girls), with a median age of 11.1 years (interquartile range, IQR, 9.8–13.9). No patients aged < 2 years underwent analysis of serum lipoprotein(a). Baseline patient characteristics are summarized in Table 1. The median age of subjects was higher for boys (median 13.5 years, IQR 10.8 to 16.2) than for girls (median 10.7 years, IQR 9.5 to 12.1, *p* < 0.01). The median lipoprotein(a) level in total subjects was 21.5 nmol/L (IQR 8.2–51.7). The median lipoprotein(a) level was slightly higher for girls (22.7 nmol/L, IQR 9.9 to 59.7) than for boys (17.7 nmol/L, IQR < 7.0 to 36.6, *p* = 0.03). The median lipoprotein(a) level was not significantly different by age group (*p* ≥ 0.05).

**Figure 1:**
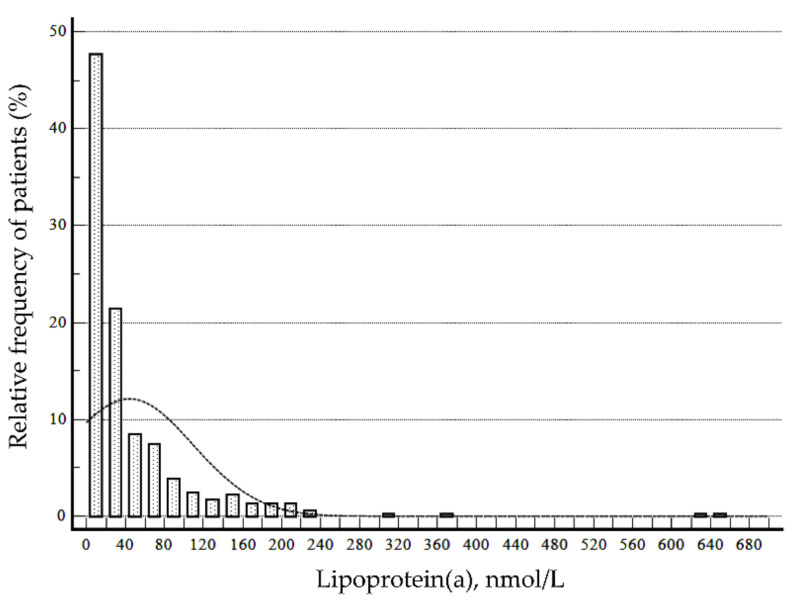
Distribution of serum lipoprotein(a) levels in a Korean pediatric population (*n* = 416).

The distribution of serum lipoprotein(a) levels is summarized in Table 2 and Figure 1. Among 416 patients, the 90th percentile value of the initial lipoprotein(a) measurement was 107.8 nmol/L. The proportion of patients with lipoprotein(a) ≥100 nmol/L was 11.3%, and the proportion of patients having extremely high lipoprotein(a) > 430 nmol/L was 0.5% (2/416). The proportion of patients with lipoprotein(a) ≥100 nmol/L was not significantly different among sex or age group (*p* ≥ 0.05).

### 3.2. Serum Lipoprotein(a) Level during Follow-Up

**Figure 2:**
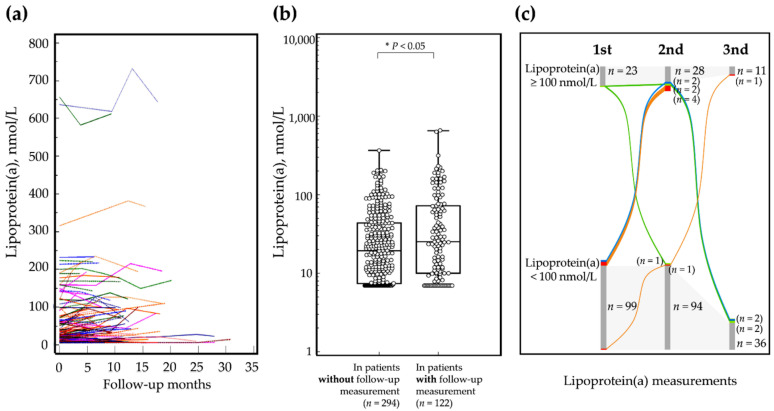
Intra-individual changes in serum lipoprotein(a): (**a**) Individual data in 122 subjects during follow-up; (**b**) Comparison of initial serum lipoprotein(a) in patients who experienced (or not) follow-up serum lipoprotein(a) levels >100 nmol/L in 416 children; (**c**) Sankey diagram showing the percentages of children that maintained stable lipoprotein(a) and that showed changes (who experienced serum lipoprotein(a) >100 nmol/L during follow-up) among 416 children. Gray flows indicate cases of stable lipoprotein(a); colored flows indicate cases that experienced changes in serum lipoprotein(a) levels during follow-up; green flows indicate cases that experienced initial serum lipoprotein(a) ≥100 nmol/L that decreased to <100 nmol/L (*n* = 2); red flows indicate cases that experienced initial serum lipoprotein(a) <100 nmol/L that increased to ≥100 nmol/L (*n* = 5); blue flows indicate cases that experienced initial serum lipoprotein(a) <100 nmol/L, that increased to ≥100 nmol/L and then decreased to < 100 nmol/L (fluctuation, *n* = 2). Number of subjects is available on the right side of the bar of each measurement.

Among all patients, 122 (29.3%, 21 boys and 101 girls) underwent at least two lipoprotein(a) measurements. The median follow-up period was 6.7 months (IQR, 5.5–11.8) and was not significantly different between boys and girls (*p* ≥ 0.05). The median lipoprotein(a) level at initial measurement was significantly higher in patients who participated in follow-up (median 25.1 nmol/L, IQR 10.0 to 72.4), than in patients without follow-up (median 19.4 nmol/L, IQR 7.4 to 43.7, *p* = 0.01) as seen in Figure 2.

Intra-individual changes in serum lipoprotein(a) are summarized in Figure 2 and ranged from −73.9 to 95.7 nmol/L, while the percentage change ranged from −54.1% to 197.6% during follow-up. Among the 122 children who had follow-up serum lipoprotein(a) results, 92 (75.4%) showed stable lipoprotein(a) <100 nmol/L, 20 (16.4%) showed stable lipoprotein(a) ≥100 nmol/L, and 10 (8.2%) experienced changes in serum lipoprotein(a) level during follow-up (three experienced initial serum lipoprotein(a) ≥100 nmol/L that decreased to < 100 nmol/L and seven experienced initial serum lipoprotein(a) <100 nmol/L that increased to ≥100 nmol/L at least once). The lipoprotein(a) level in those with an initial lipoprotein(a) <100 nmol/L, and who experienced an increase to ≥100 nmol/L, ranged from 42–98 nmol/L, with the follow-up duration ranging from 3.4–19.0 months. There were no statistical differences in age group for change of serum lipoprotein(a) >100 nmol/L, or ≥ 100 nmol/L, during follow-up (*p* ≥ 0.05).

## 4. Discussion

In this study, we retrospectively evaluated serum lipoprotein(a) test results in a Korean pediatric population that visited local clinics and hospitals, investigating the distribution and intra-individual quantitative and qualitative changes during follow-up. As there is a lack of data regarding the distribution of serum lipoprotein(a) levels in the Korean pediatric population, the results of this study will expand basic knowledge, which can be used in the diagnosis and management of dyslipidemia in Korean pediatric populations, including translational research and clinical trials.

In this study, the number of girls tested for serum lipoprotein(a) was greater than that of boys. Additionally, girls aged 9 to 11 years, which is the age range recommended for universal screening for dyslipidemia, were the most prevalent population (47.4% of total subjects). This population distribution was comparable to the proportion of patients tested for serum lipoprotein(a) between January 2019 and October 2021 (the latest month), with results available through the public database, Healthcare Bigdata Hub by Health Insurance Review and Assessment Service (HIRA, test code D2620). According to the HIRA database, 5561 patients were tested for serum lipoprotein(a) in a Korean pediatric population aged < 19 years. Within the database, information on patient age is only available for groups in 5-year intervals (<5, 5 to 9, 10 to 14, 15 to 19 years). Among a total of 5561 patients, 53.4% were female. Girls aged 10 to 14 years were the most prevalent population (34.7% of females), followed by girls aged 5 to 9 (32.2% of females, http://opendata.hira.or.kr/op/opc/olapDiagBhvInfo.do, accessed on 7 June 2022). Although there have been no studies regarding large population-based serum lipoprotein(a) data for Korean pediatric patients, the prevalence of dyslipidemia is about 6.5% for those with hypercholesterolemia (5.8% in males, 7.4% in females); 4.7% for those with high LDL (4.1% in males, 5.5% in females); 10.1% for those with high triglycerides (9.8% in males, 10.3% in females); and 11.9% for those with low HDL (14.5% in males, 9.5% in females), according to The Korea National Health and Nutrition Examination Survey IV (2007–2009). Although it cannot be clearly explained why the proportion of girls who performed the serum lipoprotein(a) test is higher than that of boys, one possibility is the pattern of medical service utilization by the guardians of Korean children. The population of this study included children who visited local clinics and hospitals and underwent serum lipoprotein(a) measurement. The public HIRA database contains all reimbursed lipoprotein(a) tests performed in local clinics, hospitals, and tertiary university hospitals, which might have different population characteristics, such as comorbidities. For example, the prevalence of precocious puberty, one reason for visiting local clinics and hospitals, is higher in girls than in boys [23]. Considering the possible association between hyperlipidemia and precocious puberty, this might result in differences between girls and boys who tested for serum lipoprotein(a) levels [24]; however, detailed clinical information was limited for this study.

In this study, lipoprotein(a) levels seemed to be stable in females; it was not possible to make such a conclusion in males because of the low number of assays in younger boys. Additionally, the number of patients aged 2 to 4 years was too small to provide representative data. The relative stable lipoprotein(a) level by age increase was inconsistent with a recent study performed in 2740 pediatric patients of European ancestry, who visited lipid clinics and underwent testing from pediatric age to adulthood [17]. They reported that lipoprotein(a) levels increased throughout the pediatric period until the age of 15, after which it seemed to stabilize among patients treated with statin and ezetimibe [17]. The differences in changes in lipoprotein(a) with age, between this study and the study by de Boer et al., may be due to differences in population characteristics of the study subjects. The study by de Boer et al. included a pediatric population that visited lipid clinics in the Amsterdam UMC, a medical center conducting complex patient care (more than two-thirds of the children had molecularly-proven heterozygous familial hypercholesterolemia) [17]. In the present study, we used data from local clinics and hospitals with limited clinical information on the reasons for lipoprotein(a) tests.

In this study, initial serum lipoprotein(a) levels did not vary according to sex and age, which was comparable to findings in previous studies in which lipoprotein(a) levels were usually determined genetically and present from birth, continuing throughout the individual’s lifetime [5,6]. In the present study, the distribution of lipoprotein(a) results at the 90th percentile value was comparable to that of previous studies performed in Asian populations [1,7]. The prevalence of patients having lipoprotein(a) ≥100 nmol/L was 11.3%, the tentative universal cut-off for increased lipoprotein(a), which seems to be appropriate as the reported prevalence of low HDL in the Korean pediatric population is 11.9% [18].

In this study, about 5.7% of patients with an initial lipoprotein(a) of <100 nmol/L experienced an increase to ≥100 nmol/L during follow-up. It has been reported that 90% of the lipoprotein(a) level is determined genetically. European guidelines recommend a single lifetime measurement [5,12,17]; however, in this study population, some patients experienced an increase to ≥100 nmol/L during follow-up, ranging from 3.4 to 19.0 months (the lowest initial lipoprotein(a) level of those patients was 42 nmol/L). These results suggest the need for follow-up measurement of lipoprotein(a) in some pediatric patients [17]. A recent study performed by de Boer et al., which included 2740 children of European ancestry who visited a pediatric lipid clinic, reported that lipoprotein(a) levels increased by 22% from the age of eight years in those who reached adulthood without medication [17]. It also suggested that measuring lipoprotein(a) more than once during childhood would reduce substantial over-or under-estimation and may possibly result in over-or under-treatment, as lipoprotein(a) increased with age and exhibited considerable variation among children (70%) [17]. The clinical significance of serum lipoprotein(a) in children with a future prediction of cardiovascular and valvular diseases should be measured over the long-term; therefore, future studies with population-level data are needed in order to clarify the significance of serum lipoprotein(a) levels in the Korean pediatric population [5,6,25].

In this study, the prevalence of extremely high lipoprotein(a) > 430 nmol/L was 0.5% of tested subjects, which was comparable to previous findings [12]. A lipoprotein(a) level > 430 nmol/L has been suggested to have an increased lifetime risk of atherosclerotic, cardiovascular and valvular diseases, which is similar to that of patients having heterozygous familial hypercholesterolemia [12,26]. In this study, the prevalence of 0.5% of subjects whose lipoprotein(a) was >430 nmol/L was comparable with the prevalence of heterozygous, familial hypercholesterolemia in previous studies [12,26,27,28,29]. Although the clinical information of patients was limited in this study, lipoprotein(a) measurement can help clinicians detect pediatric patients at high lifetime risk of cardiovascular events [6,17].

In Green Cross Laboratories, 416 patients were tested for serum lipoprotein(a) during the study period. This number corresponds about 7.5% of patients tested in Korea during a similar period according to the public database by HIRA. The strengths of this study include the large number of patients tested for serum lipoprotein(a); the distribution of lipoprotein(a) results by sex and age; intra-individual changes; and the automated immunoturbidimetric assay, traceable to IFCC SRM2B for serum lipoprotein(a) measurement.

A limitation of this study, however, was a lack of detailed clinical information associated with dyslipidemia, together with factors that might affect the significance of serum lipoprotein(a) levels, such as: familial history of dyslipidemia and lipoprotein(a); other risk factors and conditions including inflammation; other relevant laboratory findings; and medications with lipid lowering effects [6,17,19,30]. Retrospective observations, which showed that a higher proportion of girls underwent the lipoprotein(a) test than boys, may also limit the generalizability of this study. Future studies based on detailed clinical information are needed in order to clarify the clinical significance of, and intra-individual changes in, serum lipoprotein(a) levels in Korean pediatric populations.

## 5. Conclusions

In conclusion, we investigated the distribution and intra-individual changes in serum lipoprotein(a) levels in a Korean pediatric population that visited local clinics and hospitals. Intra-individual changes in serum lipoprotein(a) levels, with identification of some patients who experienced an increase in lipoprotein(a) ≥100 nmol/L, will expand our basic knowledge regarding predictive changes in serum lipoprotein(a) in Korean pediatric patients. As this study was retrospectively designed with limited clinical information, the clinical significance of this change, together with the controversies surrounding the appropriateness of repeated serum lipoprotein(a) testing in children, should be clarified using further studies of Korean populations.

## Figures and Tables

**Table 1:** Baseline characteristics of 416 Korean children and adolescents.

Characteristics		Total (*n* = 416)	Boy (*n* = 124)	Girl (*n* = 292)
Age, years (median, IQR)		11.1 (9.8 to 13.9)	13.5 (10.8 to 16.2)	10.7 (9.5 to 12.1)
Follow-up measurement, number (median, IQR)		1.0 (1.0 to 2.0)	1.0 (1.0 to 1.0)	1.0 (1.0 to 2.0)
Follow-up duration, months (median, IQR) *		6.7 (5.5 to 11.8)	6.0 (3.2 to 10.8)	6.7 (5.6 to 12.1)
Age distribution (*n*, %)	2 to 4 years	7 (1.7%)	5 (4.0%)	2 (0.7%)
	5 to 8 years	53 (12.7%)	5 (4.0%)	48 (16.4%)
	9 to 11 years	197 (47.4%)	30 (24.2%)	167 (57.2%)
	12 to 17 years	159 (38.2%)	84 (67.7%)	75 (25.7%)
Lipoprotein(a) level at initial measurement, nmol/L (median, IQR)	Total	21.5 (8.2 to 51.7)	17.7 (<7.0 to 36.6)	22.7 (9.9 to 59.7)
	2 to 4 years	14.1 (8.8 to 17.5)	14.1 (12.3 to 24.6)	11.0 (<7.0 to 15.0)
	5 to 8 years	22.0 (8.1 to 56.7)	<7.0 (<7.0 to 30.7)	22.4 (9.2 to 60.6)
	9 to 11 years	24.1 (9.8 to 57.4)	34.1 (13.1 to 83.2)	20.8 (7.9 to 51.7)
	12 to 17 years	20.5 (7.6 to 49.8)	12.8 (<7.0 to 30.6)	23.7 (11.7 to 71.7)

* Follow-up was performed in 122 children and adolescents (21 boys and 101 girls). Abbreviations: IQR, interquartile range.

**Table 2:** Distribution of serum lipoprotein(a) in 416 Korean children and adolescents.

Distribution	*n*	Mean	SD	Min	2.5th	5th	10th	25th	Med	75th	90th	95th	97.5th	Max
Total	416	43.9	65.8	<7.0	<7.0	<7.0	<7.0	8.2	21.5	51.7	107.8	165.2	200.6	656.2
Boy	124	38.7	70.2	<7.0	<7.0	<7.0	<7.0	<7.0	17.7	36.6	93.2	148.5	193.0	656.2
Girl	292	46.2	63.9	<7.0	<7.0	<7.0	<7.0	9.9	22.7	59.7	113.8	167	204.9	636.7

Abbreviations: Max, maximum; Med, median; Min, minimum; SD, standard deviation.

## Data Availability

The datasets generated and analyzed during the current study are available from the corresponding authors on reasonable request.
